# Prevalence of vaginal HPV infection among adolescent and early adult girls in Jos, North-Central Nigeria

**DOI:** 10.1186/s12879-022-07215-7

**Published:** 2022-04-05

**Authors:** Nanma T. Cosmas, Lohya Nimzing, Daniel Egah, Ayo Famooto, Sally N. Adebamowo, Clement A. Adebamowo

**Affiliations:** 1 Department of Medical Microbiology, College of Health Sciences, University of Jos, Jos, Nigeria; 2 Department of Medical Laboratory Science, College of Health Sciences, University of Jos, Jos, Nigeria; 3 Department of Medical Microbiology, Jos University Teaching Hospital, Jos, Nigeria; 4 Institute of Human Virology Nigeria, Abuja, Nigeria; 5 Department of Epidemiology and Public Health, and the Greenebaum Comprehensive Cancer Center, University of Maryland School of Medicine, 660 West Redmond Street, Baltimore, MD 21201 USA

**Keywords:** Age-specific, Prevalence, HPV, Adolescent girls, HPV vaccine introduction, Jos Nigeria

## Abstract

**Purpose:**

Knowledge of the prevalence of HPV infection among adolescent and early adult girls is essential to determining the best age for the introduction of HPV vaccine, monitoring vaccine efficacy, and giving insight into determinants of persistent high-risk HPV infection, a necessary cause of cervical cancer. Yet, there have been limited studies of HPV infection among adolescent and early adult girls in low-and-middle-income countries.

**Methods:**

In this cross-sectional study, we randomly selected 205 girls, aged 9–20 years, from 10 schools in central Nigeria. We obtained informed consent and assent, collected data, and trained participants to self-collect vaginal samples using swab stick. We genotyped HPV using SPF_10_-DEIA/LiPA_25_ and analyzed data using Stata 14®.

**Results:**

The mean (SD) age of the girls was 14.9 (2.3) years. We found HPV in 13.2% of vaginal swabs. The earliest age at which anyHPV and hrHPV infections were detected was 10 and 12 years respectively. The prevalence of any HPV peaked at 16 and 17 years, hrHPV at 16 years, lrHPV at 17 and 18 years and multiple hrHPV 18 years of age. The prevalence of hrHPV infection was 1.5% among the 9–12 years age group, 2.9% among 13–16 years and 3.4% among 17–20 years old. The commonest hrHPV types detected were 52 (3.9%), 18 (1.5%) and 51 (2.4%). The most common lrHPV types was 6 (2.9%).

**Conclusion:**

The prevalence of HPV infection in these urbanized young girls in Nigeria is high and commences after 9 years of age. HPV vaccination in this population should start at 9 years of age or younger to prevent the establishment of persistent HPV infection.

**Supplementary Information:**

The online version contains supplementary material available at 10.1186/s12879-022-07215-7.

## Key Messages

- The prevalence of HPV was 13.2% among adolescent girls in Jos, North-Central Nigeria.
- The earliest age at vaginal HPV infection was 11 years of age.
- The most predominant hrHPV type in vaginal swab was HPV 52.
- These findings point to the need for introduction of HPV vaccine starting at 9 years of age among girls in Nigeria to give maximum protection.

**Supplementary Information:**

The online version contains supplementary material available at 10.1186/s12879-022-07215-7.

## Introduction

Human papillomavirus (HPV) infection is the commonest oncogenic virus infection in the world. It is associated with cancers of the cervix, anus, vagina, oropharynx, vulva, oral cavity, penis and larynx [1, 2]. In 2018, there were estimated 569,000 new cases of cervical cancer and 311,000 deaths worldwide [3]. Individuals are exposed to HPV infection at onset of sexual maturation and acquisition of new sexual partners [4, 5]. Globally, data have shown that HPV prevalence is highest among young women of less than 25 years [6]. Although the prevalence and type distribution of HPV vary across geographical regions, the infection among adolescent girls usually peaks at around 15 and 16 years of age and coincides with the onset of sexual activities [5, 7]. Among older women, several studies have shown that HPV has a bimodal peak with the first peak seen among women younger than 30 years of age and the second one among older women greater than 45 years of age [8, 9]. Data are however scarce on the earliest age at which HPV is detected among adolescent girls and the exact age at which the first peak of HPV infection is observed.

HPV vaccination has been shown to be efficacious for prevention of infection and associated diseases [10] The vaccine induces stronger immunologic response when administered at a young age. Therefore, to prevent establishment of HPV infections and reduce the risk of associated diseases, the WHO recommends vaccination of girls, age 9–14 years, before they become sexually active [11]. The U.S. Food and Drug Administration (FDA) has licensed several prophylactic HPV vaccines. Of these, Gardasil ® 9, a nanovalent vaccine that covers HPV types 6, 11, 16, 18, 31, 33, 45, 52, and 58, licensed for use in females aged 9 to 45 years is the most widely used in the US and Australia [12, 13]. There are few studies of the epidemiology of HPV infection in young girls in low- and middle-income countries to ascertain the most appropriate age range for HPV vaccination.

Substantial progress has been made in reducing HPV prevalence in developed countries like the USA [14] and Australia [15]. By 2018, 80 countries have included HPV vaccine in their national immunization programme for girls [16]. HPV infection remains of considerable public health significance, particularly in sub-Saharan Africa (SSA) where most countries are yet to implement nationwide HPV vaccination strategies [17].

While there has been several studies of the prevalence of HPV infection among HIV positive and negative women in Nigeria [18, 19], data are scarce regarding infection among adolescent and early adult girls. In the study we report the first study of the epidemiology of HPV infection in young girls in Nigeria.

## Methodology

### Participant enrolment and sample collection

We randomly selected 15 out of 300 registered high schools in Jos, North Central Nigeria for this study and received permission to conduct the study in 10 (66.7%) of the schools. Between May and November 2016, we contacted 700 female students and their parents, of whom 232 girls and their parents agreed to participate in the study giving a 33.1% response rate. We obtained consent from the parents of girls younger than 18 years of age and assent from the girls. Older girls gave consent in their self-cognizance.

We conducted face-to-face interview with the girls in company of female nurses or researchers using structured questionnaires that covered socioeconomic factors, family characteristics, gynecologic history, sexual hygiene and practices. Questionnaire designs were guided by the research questions and literature review. The questionnaires were pre-tested on 10 individuals to evaluate their performance and corrected as required.

Prior to sample collection, we trained study participants on how to self-obtain vaginal samples using sterile swab sticks—a small cotton swab on a wooden handle packaged in an individual reclosable plastic pack. After the training, each girl was given 2 swab sticks and instructed to:

- Remove the swab from the reclosable pack.
- Gently insert the cotton swab into the vagina while squatting or standing with legs apart.
- Gently turn the swab three times and remove.
- Replace the swab in the plastic pack.
- Repeat this procedure with the second swab stick.
- Swabs were inserted for about 3 cm and NC was always present to guide the girls

The first collected vulvo-vaginal swab was immediately stored on ice pack in a cooler until transported to the Plateau State Human Virology Research Centre (PLASVIREC) sample repository for storage at −80 °C, usually within three hours of collection. The second swab was immediately applied on Hydrion pH test paper (pH 2.8–4.6, 4.5–6.1 & 5.5–8.0) (Micro Essential Lab. Inc. New York) the colour change was compared with the provided standards.

### HPV detection and genotyping

Collected samples were transported to the African Collaborative Centre for Microbiome and Genomics Research (ACCME), Institute of Human Virology, Abuja, Nigeria for HPV detection and genotyping. To prepare the vulvo-vaginal swab for DNA extraction, 1 millilitre of PBS was added to the dry swabs inside the cryovials and vortexed to dislodge the cells from the cotton wool.

DNA extraction was carried out using Cador® Pathogen 96 QIAcube® HT Kit (Qiagen, Germany) on QIAcube HT (QIAGEN, Germany). The DNA was amplified and HPV detected using SPF_10_ (DDL Diagnostic Laboratories, The Netherlands) which simultaneously detects HPV types 2, 3, 4, 5, 6, 7, 8, 11, 13, 14, 16, 18, 20, 26, 27, 28, 30, 31, 32, 33, 34, 35, 37, 39, 40, 42, 43, 44, 45, 51, 52, 53, 54, 55 (re-classified as a subtype of HPV44), 56, 57, 58, 59, 61, 62, 64 (re-classified as a subtype of HPV34), 65, 66, 67, 68, 69, 70, 71, 72, 73, 74, 75, 76, 81, 82, 83, 84, 85, 86, 87, 89, 90, 91, 95, 97, 102, 106, 114 and 115. Positive samples were tested with reverse hybridization line probe assay (LiPA_25_) which can simultaneously identify 25 HPV types (6, 11, 16, 18, 31, 33, 34, 35, 39, 40, 42, 43, 44, 45, 51, 52, 53, 54, 56, 58, 59, 66, 68–73, 70, and 74) according to the manufacturer’s (DDL Laboratories, Netherlands) instructions. This is a highly sensitive broad-spectrum PCR-based assay for qualitative detection of HPV and suitable for epidemiological studies.

### Ethical approval and informed consent

The study protocol was approved by the institutional review board of the Jos University Teaching Hospital (JUTH). Written informed consent was obtained from the parent/guardian of participants who were less than 18 year of age and assent obtained from the participants themselves. For participants who were greater than or equal 18 years of age, they provided written informed consent prior to enrolment.

### Data management and statistical analysis

We entered data into REDCap electronic database and analyzed using Stata version 14 (StataCorp, College Station, Texas USA). High-risk HPV types were defined as types 16, 18, 26, 31, 33, 35, 39, 45, 51, 52, 53, 56, 58, 59, 66, 88, 73 and 82, including the probable hrHPV types and low-risk types were defined as types 6, 11, 40, 42, 43, 44, 54, 61, 68/73, 70, 72, 74, 81 and CP6108 [20]. Univariate analysis was done using frequency distribution and proportion for categorical variables and descriptive statistics for continuous variables. Bivariate analysis to test for association was done using chi square or fisher’s exact test for categorical variables and t-test for continuous variables; means and standard deviations of continuous variables were computed. A p-value < 0.20 was used as a criterion for the inclusion of variables in the multivariable analysis. We set a *p*-value < 0.05 as statistically significant.

## Results

### Participants’ characteristics and baseline HPV prevalence

We excluded 27 participants (11.6%) because they were unable to collect vulvo-vaginal swab leaving 205 participants. The mean age (SD) of the excluded girls was 14.7 (1.97) years while that of the remaining study participants was 14.9 (2.34) years (*p*-value 0.67). Most of the excluded participants (17, 63.0%) were from public schools while the remaining ten participants (37.0%) were from private schools. About half of the remaining participants, (104/205, 50.7%), were recruited from public schools while 49.3% (101/205) were from private schools (*p*-value 0.24). There were no significant differences between girls who were unable to collect their samples and were excluded from the study and those who remained in the study.

Some 13.2% (27/205) of the girls had any HPV infection. There was no difference in type of school attended and socio-economic status of HPV positive compared with HPV negative girls. Most (145/205, 70.7%) of the girls in this study had attained menarche and there was significant difference in menarcheal status comparing HPV positive (88.9%) and HPV negative (68.0%) girls (*p* = 0.03). Only 9.3% (19/186) of the girls reported positive history of sexual intercourse and this was significantly different (*p* = 0.01) comparing HPV positive (22.2%) and HPV negative (7.3%) girls. The mean (SD) age at first sexual intercourse for girls who reported positive sexual history was 13.3 (3.58) years, and this was of marginal statistical significance comparing HPV positive (12.0(3.46)) and HPV negative (16.2(1.72) girls (*p* = 0.07). Most of the girls (57.9%, 11/19) who reported sexual intercourse first experienced it at 15 years of age or less and 15.8% (3/19) experienced it before the age of 10 years. Among HPV positive girls, most (83.3%, 5/6) of those who have ever had sex had it after 15 years of age compared to only 23.1% (3/13) of HPV negative girls (*p* = 0.04). Most of the girls with sexual experience (17/19, 89.5%) reported one or two lifetime sexual partners while two girls (10.5%) reported more than two sexual partners. All the sexually experienced HPV negative girls had one or two sexual partners while 66.7% (4/6) of the HPV positive girls had one or two sexual partners and the rest (33.3%) have had three or four sexual partners (*p* = 0.09). Of the girls who had positive sexual history, 11 (57.9%) reported that the sex was forced and without their consent but the prevalence of this not different comparing HPV positive and HPV negative girls (*p* = 1.00). Among those girls with positive sexual history who reported the age of their sexual partners, most (6/8, 75.0%) were older than 21 years of age, as were the sexual partners of all the HPV positive girls compared to only 33.3% (1/3) of the HPV negative girls (*p* = 0.11).

We did not observe any significant differences or trends in the last time the girls had sexual contact, their partner’s marital status (where known), history of masturbation, use of toys for masturbation, use of condoms, douching, and pH of the vagina comparing HPV positive and HPV negative girls. The girls in this study discussed sex more with others than with their mothers or fathers. Among the others they discussed sex with, the most common were friends, aunts, and sisters. They had low level of knowledge about HPV infection or HPV vaccination (Table 1).

**Table 1:** Characteristics of the study population and baseline HPV prevalence

Characteristic	All subjects(n = 205) No. (%) mean (SD)	HPV Negative (n = 178) No. (%) mean (± SD)	HPV Positive (n = 27) No. (%) mean (± SD)	p-values
Age (yrs)	14.9 (2.34)	14.7(2.26)	15.7 (2.66)	0.03
9–12	40 (19.5)	35 (19.7)	5 (18.5)	
13–16	115 (56.1)	105 (59.0)	0 (37.0)	
17–20	50 (24.4)	38 (21.4)	12 (44.4)	
Type of school				0.59
Public	104 (50.7)	89 (50.0)	15 (44.4)	
Private	101 (49.3)	89 (50.0)	12 (55.6)	
Socio-economic status				0.49
Low	83 (40.5)	71 (39.9)	12 (44.4)	
Middle	81 (39.5)	73 (41.0)	8 (29.6)	
High	41 (20.0)	34 (19.1)	7 (25.9)	
Menarche				0.03
No	60 (29.3)	57 (32.0)	3 (11.1)	
Yes	145 (70.7)	121 (68.0)	24 (88.9)	
Ever had sex				0.01
No	186 (90.7)	163 (92.7)	21 (77.8)	
Yes	19 (9.3)	13 (7.3)	6 (22.2)	
Age at first sex (yrs)	13.3 (3.58)	12.0 (3.46)	16.2 (1.72)	0.07
< 10	3 (15.8)	3 (23.1)	0 (0.0)	
10–12	5 (26.3)	5 (38.5)	0 (0.0)	
13–15	3 (15.8)	2 (15.4)	1 (16.7)	
16–18	8 (42.1)	3 (23.1)	5 (83.3)	
Number of sexual partners				
0	186 (90.7)	165 (92.7)	21 (77.8)	
1–2	17 (8.3)	13 (7.3)	4 (14.8)	
3–4	2 (1.0)	0 (0.00)	2 (7,4)	
Forced Sex				0.60
No	8 (42.1)	6 (46.2)	2 (33.3)	
Yes	11 (57.9)	7 (53.8)	4 (66.7)	
Age of sexual partner (yrs)				0.11
≤ 21	2 (10.5)	2 (15.4)	0 (0.0)	
> 21	6 (31.6)	1 (7.7)	5 (83.3)	
No response/don’t know	11 (57.9)	10 (76.9)	1 (9.1)	
Last time of sexual contact				0.34
< 1 year	13 (68.4)	8 (61.5)	5 (83.3)	
≥ 1 year	6 (31.6)	5 (38.5)	1 (16.7)	
Partner marital status				0.68
Single	16 (84.2)	11 (84.6)	5 (83.3)	
Don’t know	1 (5.3)	1 (7.7)	0 (0.0)	
No response	2 (10.5)	1 (7.7)	1 (16.7)	
Masturbation				0.22
No	198 (96.6)	173 (97.2)	25 (92.6)	
Yes	7 (3.4)	5 (2.8)	2 (7.4)	
Use toys for masturbation				0.50
No	6 (85.7)	4 (80.0)	2 (100.0)	
Yes	1 (14.3)	1 (20.0)	0 (0.0)	
Condom use				0.85
Always	2 (10.5)	1 (7.7)	1 (16.7)	
Most of the time	1 (5.3)	1 (7.7)	0 (0.0)	
Never	13 (68.4)	9 (69.2)	4 (66.7)	
Sometimes	3 (15.8)	2 (15.4)	1 (16.7)	
Douching				0.40
No	171 (83.4)	150 (84.3)	21 (77.8)	
Yes	34 (16.6)	28 (15.7)	6 (22.2)	
Vaginal pH	4.8 (0.47)	4.8 (0.48)	4.8 (0.43)	0.88
< 4.5	7 (3.5)	6 (3.4)	1 (3.8)	
4.5	102 (50.8)	90 (51.4)	12 (46.2)	
> 4.5	92 (45.8)	79 (45.1)	13 (50.0)	
Discussed sex with father				0.19
No	151 (86.3)	134 (87.6)	17 (77.3)	
Yes	24 (13.7)	19 (12.4)	5 (22.7)	
Discussed sex with mother				0.97
No	81 (42.0)	71 (42.0)	10 (41.7)	
Yes	112 (58.0)	98 (58.0)	14 (58.3)	
Discussed sex with others				0.12
No	56 (27.3)	52 (29.2)	4 (14.8)	
Yes	149 (72.7)	126 (70.8)	23 (85.2)	
Who discussed sex with				0.06
Aunt	38 (25.5)	33 (26.2)	5 (21.7)	
Friend	55 (36.9)	45 (35.7)	10 (43.5)	
Grandma	7 (4.7)	5 (4.0)	2 (8.7)	
Health care practitioner	1 (0.7)	0 (0.0)	1 (4.4)	
Neighbour	2 (1.3)	1 (0.8)	1 (4.4)	
Sister	28 (18.8)	27 (21.4)	1 (4.4)	
Teacher	16 (10.7)	14 (11.1)	2 (8.7)	
Guardian	1 (0.7)	0 (0.0)	1 (4,4)	
Classmate	1 (0.7)	1 (0.8)	0 (0.0)	
Knowledge about HPV				0.38
No	200 (97.6)	173 (97.2)	27 (100.0)	
Yes	5 (2.4)	5 (2.8)	0 (0.0)	
Knowledge about HPV vaccine				0.38
No	200 (97.6)	173 (97.2)	27 (100.0)	
Yes	5 (2.4)	5 (2.8)	0 (0.0)	

### Group specific prevalence of HPV

**Fig. 1:**
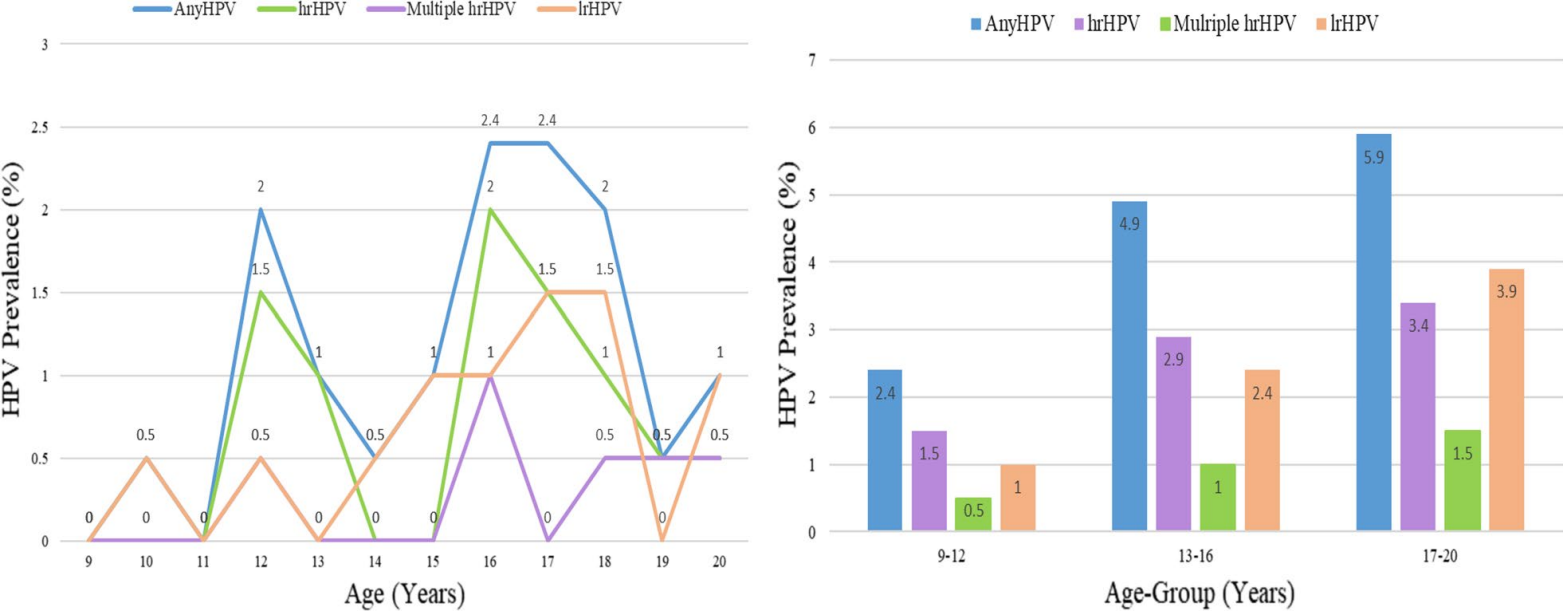
Age-specific and age group prevalence of HPV among girls, Jos, Nigeria. March 2017

The prevalence of any HPV infection in this study was 13.2% (27/205), while that of hrHPV was 59.3% (16/27), lrHPV was 55.6% (15/27), mixed high and low risk HPV was 14.8% (4/27) and multiple hrHPV was 22.2% (6/27). The age-specific and age group prevalence of any HPV among study participants are shown in Fig. 1. The first instance of any HPV infection among the girls in this study was at 10 years of age and it was a low-risk HPV infection. The first instance of high-risk HPV infection was observed at 12 years of age. The peak age prevalence of anyHPV, hrHPV, lrHPV and multiple hrHPV was 16 years of age. At this age, the prevalence of anyHPV was 6.8%, while that of hrHPV was 4.9%, lrHPV was 2.6% and multiple hrHPV was 2.4%. The prevalence of anyHPV, hrHPV, lrHPV and multiple hrHPV was highest in the 13–16 age group. The prevalence of anyHPV infection was 4.9% among 9–12 years age group, 15.1% among 13–16 years and 9.3% among 17–20 years old while that of hrHPV infection was 3.4% among 9–12 years age group, 7.8% among 13–16 years and 5.4% among 17–20 years old. The prevalence of lrHPV was 2.0% in the 9–12 years age group, 8.8% in 13–16 years age group and 5.4% in the 17 – 20 years age group while the prevalence of multiple HPV infections is 1.5% in the 9–12 years age group, 3.4% in 13–16 years age group and 2.4% in the 17–20 years age group.

### Type specific prevalence of HPV

**Fig. 2:**
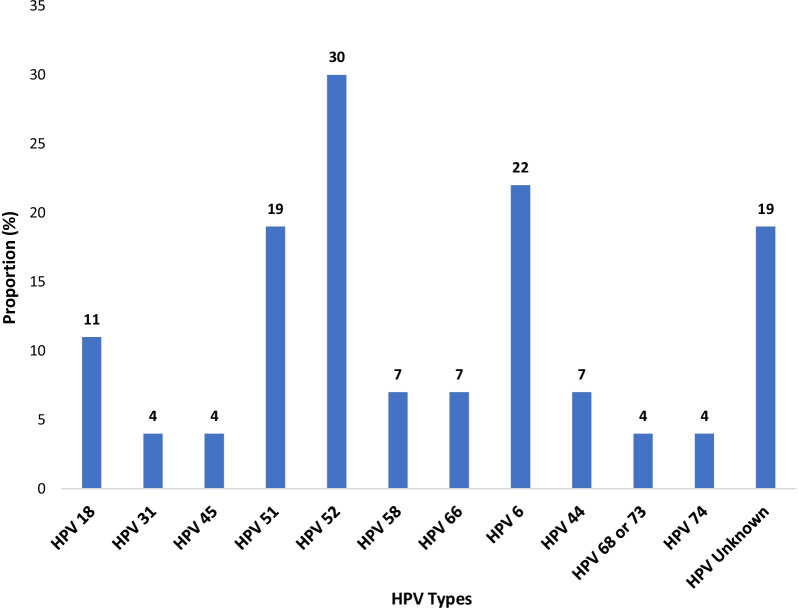
Proportion of hrHPV and lrHPV types among girls in Jos, Nigeria. March 2017

The prevalence of unknown HPV types found in this study population was 2.4%, suggesting substantial prevalence of HPV types beyond those detected using SPF_10_LiPA_25_ in this study population. We detected nine hrHPV types and the commonest hrHPV types were 52 (3.9%), 18 (1.5%) and 51 (2.4%. The other hrHPV types detected in this study were 58 (1.0%), 66 (1.0%), 45 (0.5%), and 31 (0.5%). We did not detect any HPV type 16 in this study sample. The most common lrHPV type we identified was type 6 (2.9%) followed by type 44 (1.0%). Other lrHPV types detected were types 68/73 (0.5%), and 74 (0.5). We did not detect lrHPV type 11 among this cohort of adolescent girls. The distribution of these HPV types in relation to the 27 positive participants is shown in Fig. 2.

The age at first detection of HPV types 18, 45, 66, 6, and 74 was 16 years, while HPV types 51, 52, and 58 was detected at 12 years. HPV types 31, 44 and 68/74 were first detected at 19, 15 and 17 years respectively.

### Multivariable analysis of risk factors for HPV infection

In multivariable logistic regression model that included age, ever had sex and socio-economic status, only history of ever having sex (OR (95%CI): 3.11 (1.04–9.34), *p*-value = 0.04) was significantly associated with risk of anyHPV. There was no statistically significant associations between low or high risk HPV and any other variable in multivariable analyses (Table 2).

**Table 2:** Associations between risk factors and HPV infection

Factor	Any HPV (n = 205)			hrHPV (n = 16)			Multiple hrHPV (n = 6)			lrHPV (n = 15)		
	No	OR (95%CI)	*p*	No	OR (95%CI)	*p*	No	OR (95%CI)	*p*	No	OR (95%CI)	*p*
Age-group												
9–12	40	1.00 (Reference)		3	1.00 (Reference)			1.00 (Reference)		2	1.00 (Reference)	
13–16	115	0.67 (0.12–2.08)	0.49	6	0.68 (016–2.85)	0.60		0.69 (0.06–7.82)	0.77	5	0.84 (0.72–1811)	0.86
17–20	50	2.21 (0.17–6.91)	0.17	7	2.01 (0.48–8.32)	0.48		2.49 (0.25–24.90)	044	8	3.62 (0.61–4.64)	0.12
School category												
Private	101	1.00 (Reference)		6	10 (Reference)		1	1.0 (Reference)		6	1.00 (Reference)	
Public	104	1.25 (0.55–2.82)	0.59	10	1.68 (0.59–4.82)	0.33	5	5.05 (0.58–44.01)	0.14	9	1.5 (0.51–4.38)	0.46
Socio-economic status												
Low	83	1.00 (Reference)		7	1 (Reference)		0	–	–	6	1.00 (Reference)	
Middle	81	0.65 (0.25–1.68)	0.37	5	0.71 (0.22–2.35)	0.58	4	1.01 (0.18–5.77)	0.99	5	0.84 (0.25–2.88)	0.79
High	41	1.22 (0.44–3.37)	0.70	4	1.17 (0.04–0.20)	0.81	2	–	–	4	1.39 (0.39–5.22)	0.63
Menarche												
No	60	1.00 (Reference)		2	1.00 (Reference)		0	–	–	1	1.00 (Reference)	
Yes	145	3.77 (1.09–13.00)	0.04	14	3.10 (0.68–14.08)	0.14	6			14	6.30 (081–49.07)	0.08
Ever had sex												
No	186	1.00 (Reference)		13	1.00 (Reference)		5	1.00 (Reference)		11	1.00 (Reference)	
Yes	19	3.63 (1.35–10.56)	0.02	3	2.50 (0.64–9.68)	0.19	1	2.01 (0.22–18.17)	0.53	4	4.24 (1.20–14.96)	0.03
No. sexual partners												
< 1	186	1.00 (Reference)		13	1.00 (Reference)		5	1.00 (Reference)		11	1.00 (Reference)	
1–2	17	2.42 (0.72–8.10)	0.15	2	1.77 (0.37–8.60)	0.48	1	2.26 (0.25–20.56)	0.47	3	3.41(0.85–13.66)	0.08
3–4	2	–	–	1	13.31(0.79–222.18)	0.07	0			1	15.91 (0.93–271.74)	0.56
Forced sex												
No	8	1.00 ( Reference)		0	–		0	–		2	1.00 (Reference)	0.72
Yes	11	1.71 (0.23–12.89)	0.60	3			1			2	0.67 (0,07–6.11)	
Douche												
No	171	1.00 (Reference)		13	1.00 (Reference)		5	1.00 (Reference)		11	1.00 (Reference)	
Yes	34	1.53 (0.57–4.13)	0.40	3	1.18 (.32–4.37)	0.81	1	1.01 (0.11–8.89)	1.0	4	1.94 (0.58–6.50)	0.28
Masturbation												
No	198	1.00 (Reference)		14	1.00 (Reference)		6	–	–	15	–	–
Yes	7	2.77 (051–15.04)	0.24	2	5.26 (0.93–29.58)	0.06	0			0		
Vaginal pH												
4.5	102	1.00 (Reference)		7	1.00 (Reference)		3	1.00 (Reference)		7	1.00 (Reference)	
< 4.5	7	1.25 (0.14–11.29)	0.84	0	–		0	–	–	1	0.26 (0.24–25.50)	0.48
> 4.5	92	1.23 (0.53–2.86)	0.62	9	1.47 (0.53–4.12)	0.46	3	1.11 (0.22–5.65)	0.90	6	0.95 (0.31–2.93)	0.92
Condom use												
Always	2	1.00 (Reference)	–	1	–		1			1	1.00 (Reference)	–
Most of the time	1	–		0		-	0	–	–	0	–	
Never	13	0.44 (0.02–9.03)	0.60	2	0.18 (0.007–19.56)	0.29	2			2	0.18 (0.08–4.26)	0.20
Sometimes	3	0.50 (.01–19.56)	0.71	0	–	-	0			1	0.50 (0.01–19.56)	0.71
Last time of sex												
1–5 months	5	1.00 (Reference)		1	1.00 (Reference)		1			2	1.00 (Reference)-	
0–3 weeks	4	1.50 (0.10–21.3)	0.77	1	0.13 (0.57–31.12)	0.18	0	–	–	1	0.50 (0.28–8.95)	0.28
6–11 months	4	0.50 (0.03–8.95)	0.64	1	0.13 (0.57–31.12)	0.18	0			0		
1–5 years	3	0.75 (0.34–14.97)	0.85	0	–	–	0			1	0.75 (0.38–14.97)	0.85
> 5 years	3	1	-	0	–	–	0			0		–
Discussed sex with father												
No	151	1.00 (Reference)		9	1.00 (Reference)		3	1.00 (Reference)		10	1.00 (Reference)	
Yes	24	2.07 (0.69–6.27)	0.20	4	3.16 (0.89–11.21)	0.08	1	2.14 (0.21–21.51)	0.52	1	0.61 (0.07–5.02)	0.65
Discussed sex with mother												
No	81	1.00 (Reference)		7	1.00 (Reference)		4	1.00 (Reference)		5	1.00 (Reference)	
Yes	112	1.01 (0.43–2.41)	0.97	8	0.81 (0.28–2.34)	0.70	2	0.35 (0.06–1.96)	0.23	8	1.17 (0.36–3.71)	0.79
Discussed sex anyone else												
No	56	1.00 (Reference)		2	1.00 (Reference)		0	-	–	2	1.00 (Reference)	
Yes	149	2.39 (0.78–7.20)	0.13	24	2.80 (0.62–12.74)	0.18	6			13	2.58 (0.56–11.82)	0.22
Discussed sex with who else												
Aunt	38	1.00 (Reference)	0.52		1.00 (Reference)		2	1.00 (Reference)		2	1.00 (Reference)	
Friend	55	1.47 (0.46–4.70)			1.04 (0.27–3.97)	0.95	2	0.68 (0.09–5.04)	0.71	6	2.20 (0.42–11.56)	0.35
Grandma	7	2.60 (0.40–17.48)	0.31		3.40 (0.49–23.65)	0.22	0	–	–	0	–	–
Healthcare professional	1	–	–		–	–	1	–	–	0	–	–
Neighbour	2	6.6 (0.35–123.24)	0.21		8.50 (0.44–163.89)	0.16	1	18.00 (0.80–408.07)	0.07	1	18.00 (0.80–406.07)	0.07
Sister	28	0.24 (0.27–2.22)	0.21		–	–	0	–	–	1	0.67 (0.06–7.74)	0.75
Teacher	16	0.94 (0.16–5.45)	0.94		–	–	0	–	–	2	2.57 (0.33–20.07)	0.37
Other	2	6.60 (0.35–123.34)	0.21		–		0	–	–	1	18.00 (0.80–406.07)	0.67

## Discussion

Previous studies in Nigeria have reported the prevalence of HPV infection in the general population of women and also among HIV positive women [18, 19]. Up to now, there has been few studies of the epidemiology of HPV infection among adolescent girls, who are the target group for HPV vaccination. We present here one of the first studies to focus specifically on the prevalence and type distribution of HPV infection among adolescent girls in Nigeria.

The overall prevalence of HPV found in vaginal swab in this population of adolescent females irrespective of the sample type was 13.2%. Globally, the overall prevalence of HPV infection varies according to geographical regions. Our finding is lower than the 24.5% finding among adolescent girls age 14–19 years in the USA before the introduction of HPV vaccination [21], 66.7% among girls aged 16–22 years in South Africa and 32.5% among 17–18 year old girls in Tanzania [22, 23]. These differences may be due to differences in the type of test used for the HPV detection, the age ranges, prevalence of HIV and sexual history of the participants. While other studies were among sexually active adolescent girls, our study participants were enrolled without consideration of their sexual history. Our result also differed from that obtained in a study of virgin girls in Tanzania [24]. This difference reflect differences in population prevalence of HPV. A study of adolescent and young adult girls in India [25] reported similar HPV prevalence as this study [22, 26].

We found significant associations between any HPV infection, menarcheal status and history of ever having sex. Association with age at first sex was marginally significant with most HPV positive girls having first had sex at more than 15 years of age compared with HPV negative girls, most of whom had their first sexual experience at 15 years of age or less. Age is an important determinant of HPV vaccination. The WHO recommends vaccine administration as early as age 9 years targeting girls before the onset of sexual activity. In this study, the earliest age at which anyHPV infections was detected was 11 years. HPV Infection peaked at age 16 years and declined with increasing age. This corroborates with previous findings that adolescent girls have higher exposure to HPV infection [23] and could indicate exposures to sexual activities [6, 27]. The WHO target group for the primary prevention of HPV infection is age 9–14 years, prior to sexual debut. Introduction of HPV vaccine in the population of adolescent girls in SSA will be of great benefit among 9–14 years old before infection peak, which was 16 years in this study. Early intervention through vaccine introduction and achievement of high vaccine coverage would reduce the risk of HPV infection among girls [28]. Recent study of the national HPV vaccination programe in England showed substantial reduction in incidence of cervical cancer and CIN3 in young women after the introduction of the HPV immunisation programme in England with near elimination of cervical cancer in women born since September 1, 1995 [28, 29].

The high HPV infection prevalence of 13.2% recorded in this study is of public health concern and underscores the importance of introduction of HPV vaccines among adolescents in Nigeria for the prevention of HPV infection and associated malignancies. Although more than 90% of all infection with any HPV resolve without treatment within 2 years approximately 10% of infection fail to resolve, resulting in persistent infection with the virus [30]. Persistent infection with hrHPV type is the primary risk factor for the development of HPV related cancer [31]. The finding of high prevalence of HPV infection in this study demonstrates the need for HPV persistence studies among adolescent girls to identify those at high risk of cervical cancer who require monitoring.

HPV vaccination is best administered to HPV naïve individuals and before the onset of sexual activities [11]. The earliest age for ever had sexual contact in this study was 6 years of age, And other studies have also shown that sexual debut before age 15 years was common among young people [32, 33]. Early age at sexual debut has implications for hrHPV infection [34] and has also been associated with other risky sexual behaviour [35], which contribute to increase the likelihood of HPV infection persistence and progression to cancer. Most of the first sexual experiences reported by girls in this study was forced and most of the participants have never discussed sex with either parent. The commonest persons they discussed sex with was their friend followed by aunts. This has implications for sexual health, behavior, and education of adolescent girls in Nigeria. Given the importance of sexual exposure and its characteristics as described in this study, sexual education at age 9–10 years, before the first sexual experience, is recommended to reduce risk of risky sexual behavior and associated disorders in this population.

The major limitation of the study is the small sample size which reduced the power of the analyses, particularly for exploration of associations between group and type-specific HPV infections and their risk factors. Furthermore, this study was carried out among school attending adolescent and early adult girls. The findings may not be wholly generalizable to girls outside of school settings where other factors are likely to play a role. An important avenue for future research may be to consider including girls who are out of school to give a more generalizable outcome.

## Conclusion

To the best of our knowledge, this is the first HPV study to be conducted in Nigeria focused on young girls who are the primary target group for HPV vaccine. Knowledge about HPV infection prevalence among adolescent girls had provided an important gateway into HPV vaccine introduction in and vaccine success evaluation. Our findings indicate that HPV vaccination starting at 9 year of age would adequately cover the at-risk population in Nigeria. The study lays the foundation for future monitoring of vaccine efficacy and give insight into the determinants of persistent high-risk HPV infection (hrHPV) among the study population.

## Supplementary Information

**Additional file 1.** HPV among adolescent and young adult grils_dataset.

## Data Availability

The datasets used and/or analysed during the current study are available in a Additional file 1.

## References

[1] Saraiya MS, Unger ER, Thompson TD, Lynch CF, Hernandez BY, Lyu CW (2015). US assessment of HPV Types in cancers: implications for current and 9-valent HPV vaccines. J Natl Cancer Institute..

[2] Viens LJ, Jane Henley S, Watson M, Markowitz LE, Thomas CC, Thompson TD (2016). Human papillomavirus-associated cancers—United States, 2008–2012. Morb Mortal Wkly Rep.

[3] Ferlay JF, Ervik M, Lam F, Colombet M, Mery L, Piñeros M, et al. Global cancer observatory: cancer today. Cancer Today. 2018. https://gco.iarc.fr/today.

[4] Winer RL, Lee SK, Hughes JP, Adam DE, Kiviat NB, Koutsky LA (2003). Genital human papillomavirus infection: incidence and risk factors in a cohort of female university students. Am J Epidemiol.

[5] Weaver B, Tu W, Shew M, Qadadri B, Tong Y, Denski C (2011). 2. Acquisition of first human papillomavirus infection related to first vaginal intercourse and other sexually transmitted infections in adolescent women. J Adolesc Health..

[6] Smith JS, Melendy A, Rana RK, Pimenta JM (2008). Age-specific prevalence of infection with human papillomavirus in females: a global review. J Adolesc Health..

[7] Wellings K, Collumbien M, Slaymaker E, Singh S, Hodges Z, Patel D (2006). Sexual behaviour in context: a global perspective. Lancet..

[8] Dareng EO, Adebamowo SN, Famooto A, Olawande O, Odutola MK, Olaniyan Y (2019). Prevalence and incidence of genital warts and cervical Human Papillomavirus infections in Nigerian women 11 Medical and Health Sciences 1117 Public Health and Health Services. BMC Infect Dis.

[9] Torres-Poveda K, Ruiz-Fraga I, Madrid-Marina V, Chavez M, Richardson V (2019). High risk HPV infection prevalence and associated cofactors: a population-based study in female ISSSTE beneficiaries attending the HPV screening and early detection of cervical cancer program. BMC Cancer..

[10] Joura EA, Giuliano AR, Iversen OE, Bouchard C, Mao C, Mehlsen J (2015). A 9-valent HPV vaccine against infection and intraepithelial Neoplasia in women. Obstet Gynecol Survey..

[11] World Health Organization. WHO guidance note: comprehensive cervical cancer prevention and control: a healthier future for girls and women. WHO Guidelines Note. 2013; 1–12. www.who.int.

[12] Human papillomavirus (HPV) | The Australian Immunisation Handbook (cited 2022 Feb 6). https://immunisationhandbook.health.gov.au/vaccine-preventable-diseases/human-papillomavirus-hpv.

[13] Human Papillomavirus (HPV) Vaccines—National Cancer Institute (cited 2022 Feb 6). https://www.cancer.gov/about-cancer/causes-prevention/risk/infectious-agents/hpv-vaccine-fact-sheet.

[14] Markowitz LE, Gee J, Chesson H, Stokley S (2018). Ten years of human papillomavirus vaccination in the United States. Acad Pediatrics..

[15] McGregor S, Saulo D, Brotherton J, Liu B, Phillips S, Skinner SR (2018). Decline in prevalence of human papillomavirus infection following vaccination among Australian Indigenous women, a population at higher risk of cervical cancer: the VIP-I study. Vaccine.

[16] Gallagher KE, LaMontagne DS, Watson-Jones D (2018). Status of HPV vaccine introduction and barriers to country uptake. Vaccine.

[17] Black E, Richmond R (2018). Prevention of cervical cancer in Sub-Saharan Africa: the advantages and challenges of HPV vaccination. Vaccines.

[18] Adebamowo SN, Olawande O, Famooto A, Dareng EO, Offiong R, Adebamowo CA. Persistent low-risk and high-risk human papillomavirus infections of the uterine cervix in HIV-negative and hiv-positive women. Front Public Health. 2017; 5(178). 10.3389/fpubh.2017.00178 PMC5519520 28785554

[19] Akarolo-Anthony SN, Al-Mujtaba M, Famooto AO, Dareng EO, Olaniyan OB, Offiong R (2013). HIV associated high-risk HPV infection among Nigerian women. BMC Infect Dis.

[20] Muñoz N, Bosch FX, de Sanjosé S, Herrero R, Castellsagué X, Shah KV (2003). Epidemiologic classification of human papillomavirus types associated with cervical cancer. N Engl J Med..

[21] Dunne EF, Unger ER, Sternberg M, McQuillan G, Swan DC, Patel SS (2007). Prevalence of HPV infection among females in the United States. J Am Med Assoc.

[22] Mbulawa ZZA, Van Schalkwyk C, Hu NC, Meiring TL, Barnabas S, Dabee S (2018). High human papillomavirus (HPV) prevalence in South African adolescents and young women encourages expanded HPV vaccination campaigns. PLoS ONE..

[23] Baisley KJ, Andreasen A, Irani J, Nnko S, Changalucha J, Crucitti T (2020). HPV prevalence around the time of sexual debut in adolescent girls in Tanzania. Sexually Transmitted Infect.

[24] Houlihan CF, Baisley K, Bravo IG, Kapiga S, de Sanjosé S, Changalucha J (2016). The incidence of human papillomavirus in tanzanian adolescent girls before reported sexual debut. J Adolesc Health.

[25] Sharma K, Kathait A, Jain A, Kujur K, Raghuwanshi S, Bharti AC (2015). Higher prevalence of human papillomavirus infection in adolescent and young adult girls belonging to different indian tribes with varied socio-Sexual lifestyle. PLoS ONE..

[26] Traore IMA, Zohoncon TM, Ndo O, Djigma FW, Obiri-Yeboah D, Compaore TR (2016). Research article oncogenic human papillomavirus infection and genotype characterization among women in Orodara, western Burkina Faso. Pak J Biol Sci.

[27] Tarkowski TAA, Koumans EHH, Sawyer M, Pierce A, Black CMM, Papp JRR (2007). Epidemiology of human papillomavirus infection and abnormal cytologic test results in an urban adolescent population. J Infect Dis.

[28] Schiffman M, Saraiya M (2017). Control of HPV-associated cancers with HPV vaccination. Lancet Infect Dis..

[29] Falcaro M, Castañon A, Ndlela B, Checchi M, Soldan K, Lopez-Bernal J (2021). The effects of the national HPV vaccination programme in England, UK, on cervical cancer and grade 3 cervical intraepithelial neoplasia incidence: a register-based observational study. Lancet..

[30] Stanley MA (2012). Epithelial cell responses to infection with human papillomavirus. Clin Microbiol Rev..

[31] Plummer M, Schiffman M, Castle P, Maucort-Boulch D, Wheeler C (2009). A 2-year prospective study of human papillomavirus persistence among women with a cytological diagnosis of atypical squamous cells of undetermined significance or low-grade squamous intraepithelial lesion. J Low Genit Tract Dis.

[32] Durowade KA, Babatunde OA, Omokanye LO, Elegbede OE, Ayodele LM, Adewoye KR (2017). Early sexual debut: Prevalence and risk factors among secondary school students in Ido-Ekiti, Ekiti state, South-West Nigeria. African Health Sci.

[33] Peltzer K, Pengpid S (2015). Early sexual debut and associated factors among in-school adolescents in six Caribbean countries. West Indian Med J.

[34] Bosch FX, Lorincz A, Muñoz N, Meijer CJLM, Shah KV (2002). The causal relation between human papillomavirus and cervical cancer. J Clin Pathol..

[35] Krüger-Kjær S, Van den Brule AJC, Svare EI, Engholm G, Sherman ME, Poll PA (1998). Different risk factor patterns for high-grade and low-grade intraepithelial lesions on the cervix among HPV-positive and HPV-negative young women. Int J Cancer.

